# The Intriguing Role of Hypoxia-Inducible Factor in Myocardial Ischemia and Reperfusion: A Comprehensive Review

**DOI:** 10.3390/jcdd10050215

**Published:** 2023-05-14

**Authors:** Ka-Lin Heck-Swain, Michael Koeppen

**Affiliations:** Department of Anesthesiology and Intensive Care Medicine, University Hospital Tuebingen, 72076 Tübingen, Germany; kalin.heck-swain@uni-tuebingen.de

**Keywords:** Hypoxia-inducible factors, heart ischemia-reperfusion injury, HIF stabilizers

## Abstract

Hypoxia-inducible factors (HIFs) play a crucial role in cellular responses to low oxygen levels during myocardial ischemia and reperfusion injury. HIF stabilizers, originally developed for treating renal anemia, may offer cardiac protection in this context. This narrative review examines the molecular mechanisms governing HIF activation and function, as well as the pathways involved in cell protection. Furthermore, we analyze the distinct cellular roles of HIFs in myocardial ischemia and reperfusion. We also explore potential therapies targeting HIFs, emphasizing their possible benefits and limitations. Finally, we discuss the challenges and opportunities in this research area, underscoring the need for continued investigation to fully realize the therapeutic potential of HIF modulation in managing this complex condition.

## 1. Introduction

Myocardial ischemia and reperfusion constitute significant contributors to global morbidity and mortality. Myocardial ischemia arises from the impairment of blood flow to myocardial tissue, typically resulting from intravascular thrombus formation or stenosis of a cardiac vessel. This disrupted blood flow fails to satisfy the nutrient demands of myocardial tissue, leading to a series of cellular and molecular events, such as metabolic shifts, intracellular acidosis, and reactive oxygen species (ROS) generation [1,2]. If blood flow is not promptly restored, these events can result in myocardial tissue necrosis and, ultimately, cell death.

Upon reestablishing blood flow, reperfusion injury paradoxically accelerates myocardial infarct development [3]. The complex pathophysiology involves ROS generation, calcium influx, inflammation, and microvascular dysfunction. Reperfusion can also trigger apoptosis and necrosis in cardiomyocytes, exacerbating tissue damage and impairing cardiac function. Despite these detrimental effects, reperfusion remains the primary therapeutic approach for myocardial ischemia, necessitating a deeper understanding of the underlying mechanisms to develop novel strategies that can mitigate the adverse effects while preserving its benefits.

Hypoxia-inducible factors (HIFs), a family of transcription factors, play a central role in mediating cellular responses to hypoxia [4]. Elucidating the function of HIFs in myocardial ischemia and reperfusion injury is imperative, given their potential as therapeutic targets. This review aims to scrutinize the contemporary literature on HIFs in the context of myocardial ischemia and reperfusion injury, focusing on the molecular mechanisms that regulate HIF activation and function, in addition to the downstream pathways implicated in cellular injury and protection. Furthermore, we will discuss potential therapeutic strategies targeting HIFs, underscoring their prospective advantages and limitations. Finally, we will delineate the prevailing challenges and prospects in this research area, emphasizing the necessity for continued investigation to fully exploit the therapeutic potential of HIF modulation in myocardial ischemia and reperfusion injury.

To compile the literature for this narrative review, we utilized major databases, such as PubMed, Google Scholar, and Web of Science. Given the breadth and depth of the topic, we applied several keywords and search terms, including but not limited to “Hypoxia-inducible factor,” “heart,” “myocardial ischemia,” and “reperfusion injury.” However, it is important to note that our intent was to provide a narrative review rather than a systematic one. The search terms “Hypoxia inducible factor” and “heart” retrieve approximately 4900 publications in PubMed alone, demonstrating the vastness and diversity of this field. Consequently, not all identified studies were included in this review to avoid overwhelming the reader with excessive information and to focus on the most pertinent research in this area. Therefore, this review might not cover every single study in the field but aims to provide a comprehensive overview of the most significant and relevant findings related to HIF in myocardial ischemia and reperfusion injury.

## 2. Hypoxia-Inducible Factors (HIFs)—An Overview

### 2.1. Structure of HIFs

Hypoxia-inducible factors (HIFs) represent a highly conserved family of transcription factors integral to the cellular response to low oxygen levels or hypoxia [5]. Their evolutionary conservation highlights their importance as a core biological mechanism. This is logical, considering that since the establishment of oxidative phosphorylation, which requires oxygen, an oxygen-sensing mechanism must be in place to respond when this essential metabolite becomes scarce.

Structurally, HIFs are heterodimeric proteins consisting of two subunits: An oxygen-sensitive α subunit and a constitutively expressed β subunit (also known as the aryl hydrocarbon receptor nuclear translocator or ARNT). To date, three different isoforms of the α subunit have been described: HIF1α, HIF2α, and HIF3α. While numerous studies have consistently shown the expression and identified biological functions for HIF1α and HIF2α in various tissues [6,7], the picture is less clear for HIF3α since its initial description in 2007 [8]. Studies on HIF3α show high variability across different cell types and conditions. Moreover, the tissue-expression pattern of HIF3α remains debated, as do its downstream targets. Therefore, in this review, we chose to focus on HIF1α and HIF2α.

The HIF-α subunits possess a basic helix-loop-helix (bHLH) domain and a Per-ARNT-Sim (PAS) domain, both essential for dimerization with the HIF-1β subunit, as well as an oxygen-dependent degradation domain (ODDD), determining protein stability under normoxic conditions [9,10]. The HIF-1β subunit shares structural homology with HIF-α subunits, containing a bHLH and PAS domain, but lacks the ODDD, rendering it insensitive to oxygen levels [11]. Under hypoxic conditions, HIF-α subunits stabilize and dimerize with HIF-1β, forming an active transcription factor complex. This complex binds to hypoxia-responsive elements (HREs) in the promoter regions of target genes [12,13], regulating their expression in response to changes in oxygen levels [14]. The ability of HIFs to orchestrate a wide array of cellular responses to hypoxia makes them essential players in the adaptation to low-oxygen environments, with implications for various physiological and pathological processes. 

### 2.2. Regulation of HIFs under Hypoxic Conditions

Activation of HIFs depends on post-translational modifications of the α subunit. Under normoxic conditions, HIF-α subunits are hydroxylated by prolyl hydroxylase domain-containing enzymes (PHDs) at specific proline residues within the oxygen-dependent degradation domain (ODDD) [15]. This hydroxylation results in the recognition of HIF-α by the von Hippel-Lindau (VHL) protein, a component of the E3 ubiquitin ligase complex, which targets HIF-α for proteasomal degradation [16]. Thus, during normoxic conditions, HIF-α subunits are constantly synthesized, modified, and rapidly degraded, ensuring no essential HIF-α subunits are present in the cytoplasm. In addition, factor inhibiting HIF-1 (FIH-1), an asparaginyl hydroxylase, also hydroxylates HIF-α at an asparagine residue in the C-terminal transactivation domain (CTAD) under normoxia. This hydroxylation inhibits HIF-α from interacting with transcriptional coactivators p300 and CBP [17].

This situation quickly changes when oxygen supply ceases and cells become hypoxic. The activity of PHDs is reduced due to the lack of oxygen, resulting in the stabilization of HIF-α subunits [15]. Furthermore, FIH-1 activity is suppressed, permitting HIF-α to interact with p300/CBP, which enables the HIF-dimer to promote the transcription of target genes [17]. Stabilized HIF-α subunits then dimerize with HIF-1β and translocate to the nucleus. Here, they bind to specific DNA sequences called hypoxia-responsive elements (HREs) located in the promoter regions of target genes. HREs have a consensus sequence 5’-RCGTG-3’, where R represents a purine (adenine or guanine) [12]. By binding to HREs, the HIF complex activates the transcription of target genes that participate in various adaptive responses to cope with low oxygen levels.

In summary, these oxygen-dependent regulatory mechanisms ensure a rapid and reversible response to changes in cellular oxygen levels, allowing HIFs to fine-tune the cellular adaptation to hypoxic conditions.

### 2.3. Role of HIFs in Various Physiological Processes

HIFs play a critical role in the response to hypoxia, regulating a wide range of physiological processes in different tissues and organs. In the bone marrow, HIFs promote erythropoiesis, which increases red blood cell production, facilitating oxygen delivery to tissues in hypoxic conditions [6]. In the liver, HIFs modulate glucose metabolism, increasing gluconeogenesis and glycogenolysis, leading to elevated blood glucose levels in hypoxic conditions [18]. In adipose tissue, HIFs modulate adipocyte differentiation and lipid metabolism, promoting lipolysis under hypoxia. In skeletal muscle, HIFs regulate angiogenesis, myogenesis, and glucose uptake, promoting adaptation to hypoxic conditions [19].

Furthermore, HIFs play a crucial role in regulating cell survival and apoptosis in various tissues. Under hypoxic conditions, HIFs modulate the expression of genes implicated in cell survival, preventing cell death, and promoting cell survival during ischemia [20]. HIFs also modulate inflammation and immune cell function in different tissues. Hypoxia-induced inflammation is a critical mediator of tissue injury during hypoxia, and HIFs have been shown to modulate the inflammatory response, promoting tissue repair [21].

Overall, the diverse functions of HIFs emphasize their crucial role in maintaining cellular homeostasis during hypoxia and their potential as therapeutic targets in various disease conditions, including cancer, ischemic heart disease, and pulmonary hypertension.

## 3. Myocardial Ischemia and Reperfusion Injury: Pathophysiology

### 3.1. Cellular and Molecular Events during Myocardial Ischemia

As mentioned in the introduction, myocardial ischemia leads to a series of cellular and molecular changes, including a shift towards anaerobic glycolysis, intracellular acidosis, and disturbances in ion concentrations [22,23,24]. The ischemic environment activates multiple signaling pathways in response to the stress induced by low oxygen levels. Among these pathways are those involving HIFs, which orchestrate adaptive cellular responses to hypoxia aimed at preserving cellular viability [25,26].

In the acute setting, the adaptation to ATP production under hypoxic conditions is crucial. The metabolic reprogramming favors anaerobic glycolysis to maintain cellular energy supply. In the long term, hypoxia promotes angiogenesis to improve blood flow to the ischemic tissue. However, maladaptive processes can also occur, such as the formation of connective tissue within the contractile apparatus of the heart, resulting in scar formation [27,28]. Understanding the balance between these adaptive and maladaptive responses is essential for developing effective therapeutic strategies to mitigate ischemia-induced injury and preserve cardiac function.

During myocardial ischemia, ROS, including free radicals and non-radical molecules, can cause oxidative damage to various cellular components, including proteins, lipids, and nucleic acids, exacerbating cell dysfunction and injury [29,30,31]. Elevated intracellular calcium concentrations, as a result of intracellular acidosis, impair the normal functioning of contractile proteins, leading to impaired relaxation and sustained contraction of cardiomyocytes, resulting in a decrease in their contractile function [32,33,34]. Furthermore, excessive intracellular calcium levels can activate various enzymes, such as proteases, phospholipases, and endonucleases, which may degrade cellular structures and contribute to cell dysfunction. Prolonged elevation of intracellular calcium concentrations can ultimately lead to cell death, either through necrosis or apoptosis, depending on the severity and duration of the ischemic insult.

HIFs play a crucial role in mediating adaptive responses to hypoxia, modulating gene expression, angiogenesis, and metabolic adaptation. Short-term adaptive mechanisms focus on ATP production under hypoxic conditions, while long-term mechanisms, such as angiogenesis, act in chronic ischemic conditions. Investigating the molecular mechanisms behind HIF activation, function, and their downstream pathways in the context of myocardial ischemia and reperfusion injury can provide valuable insights for therapeutic development.

**Figure 1:**
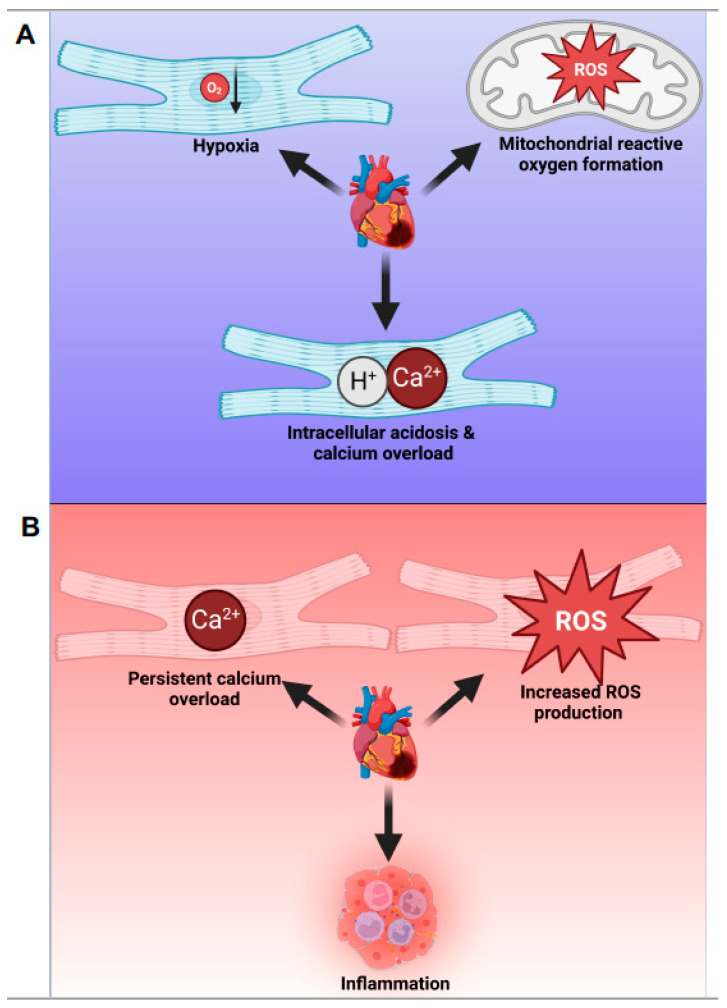
Schematic representation of key events during myocardial ischemia (left panel) and reperfusion (right panel) injury. (**A**) In ischemia, hypoxia leads to a shift towards anaerobic glycolysis, intracellular acidosis, calcium overload, and ROS generation. (**B**) During reperfusion, the reintroduction of oxygen exacerbates ROS production, calcium overload, and triggers an inflammatory response.

In summary, myocardial ischemia initiates a series of cellular and molecular changes, including a shift towards anaerobic glycolysis, intracellular acidosis, disturbances in ion concentrations, and ROS generation (Figure 1A). HIFs mediate adaptive responses to hypoxia, and understanding these events and their balance with maladaptive processes is crucial for developing therapeutic strategies for ischemia-related diseases.

### 3.2. Cellular and Molecular Events during Reperfusion Injury

Despite the detrimental effects of myocardial ischemia, the reestablishment of blood flow to the ischemic myocardium can paradoxically exacerbate tissue injury—a phenomenon known as reperfusion injury [35]. During reperfusion, the reintroduction of oxygen to the ischemic tissue leads to rapid and substantial production of ROS, causing oxidative stress and subsequent cellular damage, such as lipid peroxidation, protein oxidation, and DNA damage [36]. Calcium overload, which occurs during ischemia, can worsen during reperfusion, leading to mitochondrial dysfunction, swelling, and permeability transition pore opening [37]. This mitochondrial dysfunction triggers the activation of cell death pathways, such as apoptosis and necrosis, contributing to further tissue damage [38,39]. In addition to oxidative stress and calcium overload, reperfusion injury is characterized by an inflammatory response involving the activation of resident immune cells, such as neutrophils and macrophages, and the release of pro-inflammatory cytokines [27]. This immune response exacerbates tissue damage and can lead to the extension of the infarcted area [36,40]. The role of HIFs in the context of reperfusion injury is also an area of interest for developing novel therapeutic strategies.

Reperfusion activates the complement system, promotes the recruitment and activation of inflammatory cells, such as neutrophils and macrophages [41,42], and induces the production of cytokines, chemokines, and proteases that can exacerbate tissue injury [43]. The activation of the endothelium and the expression of adhesion molecules during reperfusion contribute to increased vascular permeability, edema formation, and leukocyte infiltration into the injured tissue, exacerbating the inflammatory response [44,45]. Additionally, the production of vasoactive mediators during reperfusion can lead to microvascular dysfunction and impaired blood flow, further aggravating tissue injury.

In summary, understanding the complex interplay of cellular and molecular events during ischemia and reperfusion is critical for developing therapeutic strategies to mitigate myocardial injury and improve patient outcomes (Figure 1B). Identifying key modulators, including HIFs and their target genes, may provide new targets for intervention, helping to minimize reperfusion injury and preserve myocardial function.

## 4. Role of HIFs in Myocardial Ischemia and Reperfusion Injury

### 4.1. Activation of HIF-Isoforms during Ischemia and Reperfusion

HIFs play a critical role in the cellular response to hypoxia during myocardial ischemia and reperfusion injury. During myocardial ischemia and reperfusion, both of HIF-α subunits, HIF-1α and HIF-2α, are stabilized. Interestingly, even though both isoforms share a high level of similarities in their protein structure and are expressed in cells side-by-side [6], and both bind to the same consensus region in the promoter of genes [46], they are non-redundant in the setting of myocardial ischemia and reperfusion. In fact, they accumulate in a heterogeneous pattern within the cells and within the myocardium.

#### 4.1.1. HIF-1α

HIF-1α, the principal orchestrator of the hypoxic response, plays a crucial role in fostering cell survival and tissue repair during ischemia [11]. Its expression is elevated in various cell types and regulates genes associated with angiogenesis, erythropoiesis, and metabolic adaptation [47]. Intriguingly, recent evidence emphasizes the significance of HIF-1α’s function in neutrophils during myocardial ischemia and reperfusion injury [48]. Neutrophils are immune cells that participate in the inflammatory response and can aggravate tissue damage.

HIF-1α exhibits a dual role in neutrophils depending on the context. In certain cases, it fosters their activation, which boosts migration, phagocytic capacity, and the production of pro-inflammatory cytokines and reactive oxygen species [49]. This activation contributes to the tissue damage and inflammation observed in myocardial ischemia and reperfusion injury. In contrast, under specific circumstances, HIF-1α can suppress neutrophil activation, acting as a protective mechanism to restrain excessive inflammation and tissue damage [50,51]. HIF-1α can diminish neutrophil activation by inducing the expression of anti-inflammatory factors, such as IL-10, which helps to resolve inflammation [52]. HIF-1α has been demonstrated to regulate neutrophil survival and enhance innate immune function [53]. However, under hypoxia, neutrophils display impaired respiratory burst activity due to increased HIF-1α protein levels [53]. Activation of HIF-1α can impede inflammation resolution by reducing neutrophil apoptosis and reverse migration [49]. In Glycogen Storage Disease Type Ib (GSD-Ib) patients, immune function is impaired due to neutrophil dysfunction, leading to increased susceptibility to bacterial infections and chronic inflammation. In these patients, neutrophils exhibit HIF-1α upregulation in the context of defective cellular energetics, potentially contributing to neutrophil dysfunction [54]. Inhibition of neutrophil function by peroxisome proliferator-activated receptor-gamma (PPAR-γ) is mediated through HIF-1α signaling [53]. When HIF-1α activity is inhibited pharmacologically, neutrophil function is restored, underscoring the relevance of HIF-1α in such settings [53]. These findings underscore the importance of HIF-1α for neutrophil function.

Neutrophils are renowned for their short lifespan and undergo programmed cell death shortly after activation. HIF-1α modulates neutrophil survival by adjusting the expression of genes implicated in apoptosis and cell survival pathways, potentially extending their lifespan and influencing the local inflammatory response [55]. Recent studies suggest that HIF-1α in neutrophils mitigates myocardial injury during acute myocardial ischemia and reperfusion by inducing the expression of the neuronal guidance protein netrin-1 [48]. This protein modulates signaling via the adenosine receptor A2b [56], which has been demonstrated in numerous studies to possess potent tissue-protective properties [57].

Beyond directly influencing inflammation by altering neutrophil behavior, HIF-1α also plays a role in the intracellular regulation of inflammation through the inflammasome. In hypoxic environments, HIF-1α regulates the expression of NLRP3, a component of the inflammasome, which influences the progression of various diseases, ranging from thromboembolism to acute lung injury [58,59,60,61,62].

HIF also plays a significant role in heart disease, facilitating the shift from fatty acids to glucose metabolism during pathological cardiac hypertrophy [63]. Furthermore, it enhances the self-catabolic process of autophagy, protecting cardiomyocytes from hypoxic-ischemic injury [64]. It is also involved in the protective response to myocardial hypoxic conditions via interaction with the iNOS gene [65].

HIF1a also mediates cardioprotective effects by reducing infarct size and injury, especially under hyperlipidemic conditions [66]. The protective mechanisms are linked to the induction of cardioprotective molecules, such as iNOS, HO-1, and EPO, which alleviate myocardial damage [67]. Additionally, stabilization of HIF-1α can confer cardioprotection against acute ischemia and reperfusion injury by preventing the opening of the MPTP [68].

HIF and its associated pathways have been found to be significant in ischemia and reperfusion, especially in aging and disease populations. HIF-1α upregulation under hypoxic conditions orchestrates adaptive responses, potentially reducing cellular damage induced by ischemia, as seen in acute myocardial infarction [69,70,71,72,73,74].

HIFs are pivotal in metabolic and glycolytic processes, especially under diabetic conditions, and can protect against organ damage, including the heart [75,76,77]. However, it is important to note that the role of HIF can change with age and under specific conditions such as diabetes, indicating that optimal HIF-1α levels could be crucial for heart health [78,79]. These findings support further investigation into HIF modulation as a therapeutic strategy.

**Figure 2:**
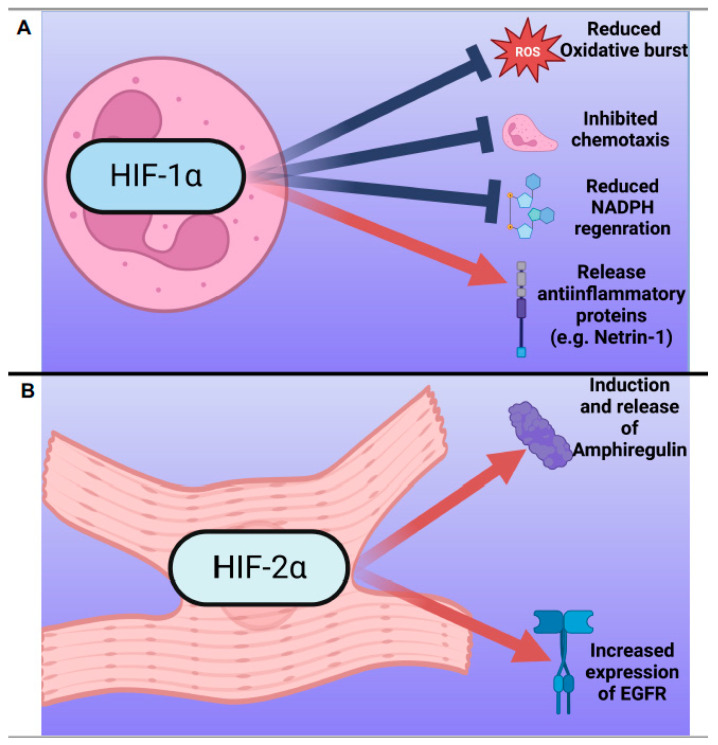
Overview of HIF-1α and HIF-2α involvement in myocardial ischemia and reperfusion injury. (**A**) Panel 1 illustrates HIF-1α stabilization in neutrophils during myocardial ischemia and reperfusion. The stabilization of HIF-1α in neutrophils initiates various cellular processes, such as reducing oxidative burst, inhibiting chemotaxis, and decreasing NADPH regeneration necessary for NADPH oxidase function. Concurrently, HIF-1α triggers an increase in Netrin-1 release, which possesses anti-inflammatory properties. These events collectively restrain excessive inflammation and tissue damage. (**B**) Panel 2 demonstrates the function of HIF-2α in cardiomyocytes, stimulating the production and release of the epithelial growth factor amphiregulin. Concurrently, HIF-2α enhances the expression of the Amphiregulin receptor, EGFR. This coordinated response results in an increase in survival kinase activation. For further details, see the main text.

In summary, HIF-1α critically influences various aspects of neutrophil function, which is pivotal in the context of myocardial ischemia and reperfusion. It can both foster and suppress neutrophil activation, depending on the context, affecting inflammation and tissue damage. HIF-1α can potentially extend neutrophil lifespan by adjusting apoptosis and cell survival pathways. Further research is needed to fully understand the mechanisms involved in HIF-1α’s nuanced regulation of neutrophil function and its effects on the inflammatory response after myocardial ischemia (Figure 2A). Recent studies suggest HIF-1α’s involvement in mitigating myocardial injury and in the progression of heart failure, emphasizing the need to explore HIF modulation as a therapeutic approach. Understanding the intricacies of HIF-1α’s role in neutrophil function may provide valuable insights for the development of novel therapeutic approaches targeting HIF-1α to mitigate inflammation and tissue damage in ischemic heart diseases.

#### 4.1.2. HIF-2α

In contrast to the more universally expressed HIF-1α, HIF-2α is selectively expressed in specific cell types and exhibits a gradual, sustained accumulation during hypoxia [9,80]. It has been identified as a key factor in safeguarding the heart during myocardial ischemia and reperfusion injury [81]. In contrast to the extensively researched HIF-1α, which has been the focus of numerous myocardial ischemia studies, investigations into HIF-2α’s role in myocardial ischemia are comparatively limited. In a recent study exploring the effects of a high-fat high-fructose (HFHF) diet on cardiac health, it was observed that 12 weeks of an HFHF diet led to a slight but not significant reduction in HIF-2α expression in heart extracts. Interestingly, under ischemia/reperfusion (IR) injury, hearts of HFHF diet mice showed a reduced expression level of HIF-2α compared to control mice [82].

Amid the acute phase of myocardial ischemia and reperfusion, HIF-2α facilitates the relaxation of existing blood vessels, augmenting blood flow to oxygen-starved regions by modulating genes implicated in vasodilation [83,84]. HIF-2α is also instrumental in vascular functions and angiogenesis, vital for blood flow regulation [85]. Research has discovered that HIF-2α, alongside HIF-1α, activates the expression of target genes modulating vascular functions in endothelial cells [83]. Overall, HIF-2α plays a critical role in blood flow regulation through its influence on vasodilation, vascular functions, and angiogenesis [85]. Evidence suggests that HIF-2α enhances blood flow to ischemic myocardium, mitigating the risk of further damage by regulating blood flow through its effects on vascular functions and angiogenesis [85,86]. In cardiomyocytes, HIF-2α controls distinct target genes, such as amphiregulin, an activator of the epithelial growth factor receptor (EGFR), which may differ from those governed by HIF-1α [81]. By inducing amphiregulin and its receptor [87], HIF-2α initiates the activation of survival kinases, boosting cell survival prospects by modulating cell metabolism. Intriguingly, HIF-2α does not seem to play a significant role in neutrophils during myocardial ischemia and reperfusion, as recent studies have demonstrated that tissue-specific deletion of HIF-2α in neutrophils does not impact infarct sizes [48].

In conclusion, HIF-2α contributes to cardioprotection during myocardial ischemia and reperfusion by fostering angiogenesis and vasodilation in endothelial cells and cardiomyocytes, while its influence on neutrophils remains limited. Recent findings indicating that diet-induced metabolic stress can influence its expression further highlight the complexity of HIF-2α’s roles and underline the need for additional research [82]. Further comprehension of HIF-2α’s functions could guide the development of innovative therapeutic strategies for heart protection (Figure 2B).

### 4.2. Differential Sensitivity of HIF-1α and HIF-2α to Oxygen Partial Pressure in Myocardial Ischemia and Reperfusion

While both HIF isoforms can bind to the same consensus region in the promoter region, they function in a non-redundant manner. The level of cellular specificity achieved is not completely clarified to date. Oxygen partial pressure plays a crucial role in the stabilization and activation of HIF-α subunits. HIF-1α and HIF-2α exhibit different sensitivities to changes in oxygen partial pressure, allowing them to mediate distinct cellular responses under various hypoxic conditions [18,80,88,89]. This differential sensitivity enables a more fine-tuned adaptation to the varying levels of oxygen availability in the ischemic myocardium. For instance, studies in different cell types have demonstrated that HIF-1α is stabilized and active under more severe hypoxia, whereas HIF-2α is stabilized and active under milder hypoxia or even in response to fluctuations in oxygen levels [90,91,92,93]. This difference in sensitivity suggests that HIF-2α might act as a slower adaptive mechanism, while HIF-1α serves as an emergency response to increase cell survival chances.

Our research supports this notion: HIF-1α stabilization and induction of Netrin-1 reduces post-ischemic inflammation in a rapid response [48], whereas HIF-2α elicits growth factors that stimulate survival kinases and have been implicated in wound healing in other contexts or disease models [81]. Additional research exploring the effects of different oxygen partial pressures on the stabilization of HIF-1α and HIF-2α in cardiomyocytes would provide valuable insights into their specific roles and potential therapeutic implications for myocardial ischemia and reperfusion injury. However, it is important to note that results regarding the differential sensitivity of HIF-1α and HIF-2α to oxygen partial pressures are not consistent across all studies [18,89]. For example, PHD2 has relatively more influence on HIF-1α than HIF-2α, and PHD3 has relatively more influence on HIF-2α than HIF-1α, which can result in the stabilization and activation of HIF-2α at higher oxygen tensions than HIF-1α [18]. Further research is needed to fully understand the differential sensitivity of HIF-1α and HIF-2α to oxygen partial pressures and to determine their specific roles in various contexts and disease models, such as non-small cell lung carcinoma cells [89], cervical cancer cells [94], and acute myeloid leukemia [95].

In summary, HIF-1α and HIF-2α, while both involved in cellular responses to hypoxia during myocardial ischemia and reperfusion, exhibit differences in expression patterns, sensitivities to oxygen partial pressure, and target gene regulation. Both HIF-1α and HIF-2α play crucial roles in cardioprotection by regulating distinct sets of genes involved in processes such as angiogenesis, vasodilation, inflammation, and cell survival.

## 5. Pharmacological Modulation of HIFs in Myocardial Ischemia and Reperfusion Injury

### 5.1. HIF Stabilizers in Clinical Trials

As previously discussed, HIF isoforms play a critical role in providing cardioprotection during myocardial ischemia and reperfusion injury. Although numerous experimental studies and some clinical works have demonstrated these protective effects, there is currently no established therapy utilizing this system to treat patients with myocardial ischemia and reperfusion. HIF stabilizers, a class of drugs that inhibit the degradation of HIF-α isoforms by targeting the prolyl hydroxylase domain (PHD) system, lead to the chronic stabilization and activation of HIF isoforms. The main goal in clinical development of HIF inhibitors was new treatment strategies for anemia due to renal failure. Various substances have been in clinical development as potential HIF stabilizers, but many have experienced setbacks due to unexpected side effects, such as severe liver failure in some cases. Nevertheless, two substances have been approved by the regulatory bodies in the United States and Europe, respectively. A seminal year in the introduction to HIF inhibitors in the clinic was the year 2021, when the results of two major clinical trials on substances from the class of HIF inhibitors were published. Two major trials investigated the role of HIF inhibitors vadadustat in patients with renal failure and subsequent anemia in non-dialysis dependent [96] or dialysis-dependent patients [97] for the treatment of renal anemia. In a pooled safety population analysis from the two clinical trials, 1739 patients received vadadustat, and 1732 patients received the control substance (darbepoetin alfa). Major adverse cardiovascular events (MACE) occurred in 22.0% of the vadadustat group and 19.9% of the control group. The expanded MACE occurred in 25.9% of the vadadustat group and 24.5% of the control group. No significant differences were observed in time to cardiovascular death or death from any cause between the two groups. The authors concluded that concerning cardiovascular safety in HIF stabilizer clinical trials, vadadustat did not meet the predefined noninferiority criterion compared to controls. The criterion was based on a composite of death from any cause, nonfatal myocardial infarction, or nonfatal stroke. Based on these results, the Federal Drug Agency in the United States did not approve vadadustat. The approval by the European Medicines Agency (EMA) is pending to date.

In the same year as the above-mentioned trials, two studies were published in the New England Journal of Medicine, investigating the efficacy of another HIF inhibitor, roxadustat, for treating anemia in chronic kidney disease (CKD) patients. Chen et al. (2019) studied roxadustat’s impact on long-term dialysis patients, while Akizawa et al. (2019) focused on CKD patients not receiving dialysis [98,99]. Both trials reported cardiovascular outcomes, but their primary objective was anemia treatment. In Chen et al.’s study, roxadustat demonstrated a lower incidence of MACE (major adverse cardiovascular events) at 13.5% compared to 14.7% in the control group. However, this difference was not statistically significant. Meanwhile, Akizawa et al. found no significant difference in MACE between roxadustat and the control group, with 12.1% in the roxadustat group and 11.3% in the control group. These trials were not designed specifically to assess cardiovascular safety, and further research is needed to draw more definitive conclusions on the differences in MACE between HIF inhibitors and conventional erythropoiesis-stimulating agents. Roxadustat has been approved for therapy in China and Europe, while approval in the United States is still pending due to safety concerns by the FDA.

In the same year as the vadadustat trials, the New England Journal of Medicine published two studies investigating the efficacy of daprodustat, another HIF inhibitor, for treating anemia in chronic kidney disease (CKD) patients undergoing dialysis. The study, conducted from 23 November 2016 to 10 August 2018, enrolled 2964 patients and compared daprodustat with erythropoiesis-stimulating agents (ESAs) for cardiovascular safety. The primary safety outcome, major adverse cardiovascular events (MACE), occurred in 25.2% of patients in the daprodustat group and 26.7% in the ESA group, demonstrating non-inferiority with a hazard ratio of 0.93 (95% CI, 0.81 to 1.07). However, the study did not find significant superiority for daprodustat concerning the three principal secondary cardiovascular outcomes, including MACE, MACE or thromboembolic events, and MACE or hospitalization for heart failure. The incidence of death from any cause was similar in both groups. Although the study focused primarily on anemia treatment, the cardiovascular safety outcomes indicate that daprodustat demonstrated noninferiority compared to ESAs in patients with CKD undergoing dialysis.

The novel group of therapeutics known as HIF stabilizers did not demonstrate a significant impact on the occurrence of MACE in the larger patient population. It is crucial to recognize that these medications were specifically designed and tested for long-term treatment rather than acute therapeutic intervention. Consequently, the potential benefits of acute administration of these drugs in reducing ischemic events remain uncertain, especially for patients undergoing planned or semi-planned ischemia and subsequent reperfusion of the heart. Examples of such patients include those undergoing cardiopulmonary bypass for heart surgery or other similar procedures. Additionally, based on the pre-clinical and basic science data previously mentioned, HIF stabilizers could potentially provide beneficial effects for patients who have experienced a myocardial infarct and subsequently undergone left heart catheterization and reopening of cardiac vessels. This suggests that HIF stabilizers may have a broader application in cardiovascular medicine beyond their current use in treating chronic renal anemia.

At present, a clinical trial investigating the use of HIF stabilizers in various off-label indications in the setting of myocardial ischemia and reperfusion (clinicaltrials.gov-ID NCT04803864). This trial investigates the efficacy and safety of early, short-term roxadustat administration for acute ST-elevation myocardial infarction patients, aiming to determine its potential in reducing infarct size and improving prognosis. The outcomes of these trials will help determine if these substances have a future in this field of medicine or if researchers need to continue exploring other substances that can stabilize HIF effectively. As the pharmacological modulation of HIF still poses several challenges, it is essential to thoroughly investigate the potential benefits and drawbacks of these compounds to optimize their use in treating cardiovascular conditions associated with ischemia and reperfusion injury.

### 5.2. Challenges and Limitations of Pharmacological HIF Modulation

Despite the potential cardioprotective effects of HIF stabilizers, there are several concerns and challenges associated with their use in treating myocardial ischemia and reperfusion injury. One major concern is the possibility of unintended side effects resulting from the chronic stabilization and activation of HIF-α isoforms. As HIFs play a role in regulating numerous cellular processes, excessive or prolonged activation may lead to undesirable outcomes such as uncontrolled angiogenesis, excessive inflammation, or metabolic imbalances.

Another issue with HIF stabilizers is their lack of isoform specificity. As HIF-1α and HIF-2α have distinct roles and sensitivities to oxygen partial pressure, the use of non-specific HIF stabilizers may lead to the simultaneous activation of both isoforms, potentially disrupting the fine-tuned balance between their functions [100,101,102]. This could result in unpredictable or counterproductive effects in the context of myocardial ischemia and reperfusion injury.

For instance, while HIF-1α activation may promote immediate cellular survival mechanisms, HIF-2α activation in regions with milder hypoxia could contribute to longer-term adaptive responses. By indiscriminately activating both isoforms, non-specific HIF stabilizers could inadvertently exacerbate the injury or hinder the tissue’s ability to recover from ischemic stress. Furthermore, HIF-1α and HIF-2α may have overlapping target genes or compete for similar binding sites, which could also lead to unintended consequences when both isoforms are simultaneously stabilized and activated.

Additionally, the optimal timing for HIF stabilizer application, as well as the long-term safety and efficacy of these drugs in patients, have yet to be fully established. While some experimental and clinical studies have shown promising results, further research is required to determine the best dosing, treatment duration, and suitable patient populations for these therapies. There is also a need to develop biomarkers to identify patients who may benefit the most from HIF stabilization and to monitor their response to treatment.

In summary, while HIF stabilizers offer potential therapeutic benefits in myocardial ischemia and reperfusion injury, significant challenges remain in understanding their precise mechanisms of action, optimizing their use, and ensuring their safety and efficacy in patients. Ongoing research and clinical trials are crucial for overcoming these hurdles and unlocking the full potential of HIF-targeting therapies in the management of myocardial ischemia and reperfusion injury.

## 6. Conclusions

Myocardial ischemia and reperfusion injury are complex pathophysiological processes that involve multiple cellular and molecular pathways. Among these, HIFs have emerged as critical mediators of cardioprotection, promoting cellular responses such as angiogenesis, vasodilation, metabolic adaptation, and anti-inflammatory effects. Oxygen partial pressure plays a crucial role in the stabilization and activation of HIF-α subunits, with HIF-1α and HIF-2α exhibiting different sensitivities to changes in oxygen levels, allowing them to mediate distinct cellular responses. While HIF stabilizers hold promise as a potential therapy for myocardial ischemia and reperfusion injury, there remain several challenges and limitations to their use, including lack of isoform specificity and unknown long-term safety and efficacy. Future research is needed to address these issues and develop effective HIF-targeting therapies that can improve patient outcomes. Additionally, a better understanding of the underlying mechanisms of reperfusion injury, including oxidative stress, calcium overload, and inflammatory response, will be critical in the development of novel therapeutic approaches for this complex condition.

## Data Availability

Not applicable.
